# When fiction meets reality: ethnofiction as a way of constructing youth narratives about delinquent paths

**DOI:** 10.3389/fsoc.2024.1456601

**Published:** 2025-04-10

**Authors:** Haydée Caruso

**Affiliations:** Department of Sociology, University of Brasilia, Brasília, Brazil

**Keywords:** ethnofiction, youth trajectories, juvenile centers, masculinities, Portugal

## Abstract

This article explores an ethnographic experience built with Portuguese young people in juvenile detention centers, using ethnofiction as a methodological strategy for approaching the public under investigation. The study examines the narratives that these young people constructed through a fictional character called João, whose experiences reflect the participants’ life stories and the paths that led them to commit crimes. The ethnofictional experimentation was developed during fieldwork in six Juvenile Detention Centers in Portugal in 2022 as part of the research project X-MEN—Masculinities, Empathy, and Non-violence, coordinated by the Center for Social Studies at the University of Coimbra. This article explores dimensions of young people’s identities and belonging, offering an in-depth view of their experiences and perspectives.

## 1 Introduction

“*Each story is unique, but some illustrate standard scripts, patterns, which crystallize processes and illustrate this or that aspect of the phenomenon studied.”* (de Gaulejac, 1996, p. 21)

One day, João entered the Juvenile Detention Center. Does anyone know him? Have you seen him around? We know very little about him at the moment. What we do know so far is that he is 15 years old and has been serving a severe sentence that has led him to remain in a closed regime for almost a year.

From this fragment of a story I told to a group of young people selected to participate in focus groups during my research in juvenile detention centers in Portugal, I began what I will refer to here as an *ethnographic experiment inspired by ethnofiction*.

It is important to note that there was already a space of trust between the researchers^1^ and the research subjects. Before this moment arrived, the young people already knew who we were and the purpose of the research we were carrying out.

They had already voluntarily answered an extensive questionnaire and participated in individual interviews. This prior knowledge of each other would be fundamental to the proposal that was to follow.

So what was proposed? After the initial dramatization of João’s life story, I, as moderator of the focus groups, informed the participants that, from that moment on, they would jointly construct everything about this fictional character’s life and finally record it in text and audio.

I can assert that this was the moment when fiction meets reality, which is why this article bears this title. The imagination, stimulated through the storytelling produced and guided by the research subjects, becomes the protagonist of the research rather than merely a set of questionnaires and interview guides that, while undoubtedly essential for conducting empirical research, often prove insufficient and restrictive in capturing the nuances of what one wants (and is able) to say about oneself.

In the research conducted, the questionnaires, individual interviews, and focus groups played a central role in constructing a comprehensive overview of the socioeconomic, cultural, and educational conditions of the young people in Juvenile Detention Centers in Portugal. However, the incorporation of ethnofiction emerged as the key element to explore these youths’ subjective dimensions and lived experiences, often underexpressed or silenced by other data collection methods.

In this case, the research dealt directly with young people shaped by criminal trajectories, making speaking in the first person particularly challenging. When recounting their experiences, participants often confront the pain and suffering associated with those experiences, which can generate feelings of shame (de Gaulejac, 1996), which is rarely spoken about, especially in front of others.

de Gaulejac points out that “shame is a painful and sensitive feeling about which it is preferable not to speak” (de Gaulejac, 1996, p. 17), warning us that this feeling tends to generate silence and withdrawal, which often leads to discomfort, misunderstanding, and loneliness. Given the objective conditions of deprivation of liberty in which the research subjects were situated, the manifestation of shame was evident, as described, expressed through their gaze, withdrawn postures, and reluctance to expose themselves or draw attention to themselves. The methodological approach adopted in this research will be examined to address the empirical challenges that studies of this nature present and to demonstrate how inspiration drawn from ethnofiction emerged as a privileged means of accessing young people’s narratives about their lives, including the trajectories that led them to experience restrictive measures of liberty.

The article is organized into four parts. The first presents the empirical context of the research and some figures that give an account of the universe of young people we came into contact with. The second part is a brief review of the literature dedicated to exploring the contributions of ethnofiction as a theoretical-methodological strategy that promotes dialog between the fictional and documentary language of cinema and the ethnographic approach from an anthropological perspective to shed light on the events, people, and cultures that we want to describe and understand.

The third is dedicated to reflecting—in methodological and ethical terms—on using the fictional resource of verisimilar elements in the life stories of the young people surveyed as a strategy to enter the different social worlds of which they are part. In this case, talking about themselves through the voice and life of another imaginary character was the guiding thread for exploring the nuances of their trajectories, which are sometimes very difficult to relate in the first person, as argued above. More than the search for singularities in the life stories, the methodology employed contributed to mapping patterns associated with the multiple economic, educational, cultural, and socio-emotional vulnerabilities experienced by these young people.

In the fourth and final part, the results of this experiment are presented, as well as the analysis of the findings. The central points they chose to value in each story are how they write, the words they use, and how the youth express their ideas to be explored. It is essential to understand the profile of the young people who are currently in juvenile detention centers in Portugal, their conceptions of gender, masculinities, and identities, and how the possible absence of care in their lives—read here as family, community, and institutional support networks—has led to the construction of forms of belonging in which violence is simultaneously configured as a language and a mechanism for insertion into the world (Machado da Silva, 2011; Machado da Silva and Menezes, 2019).

## 2 The context of the empirical research

**Figure 1:**
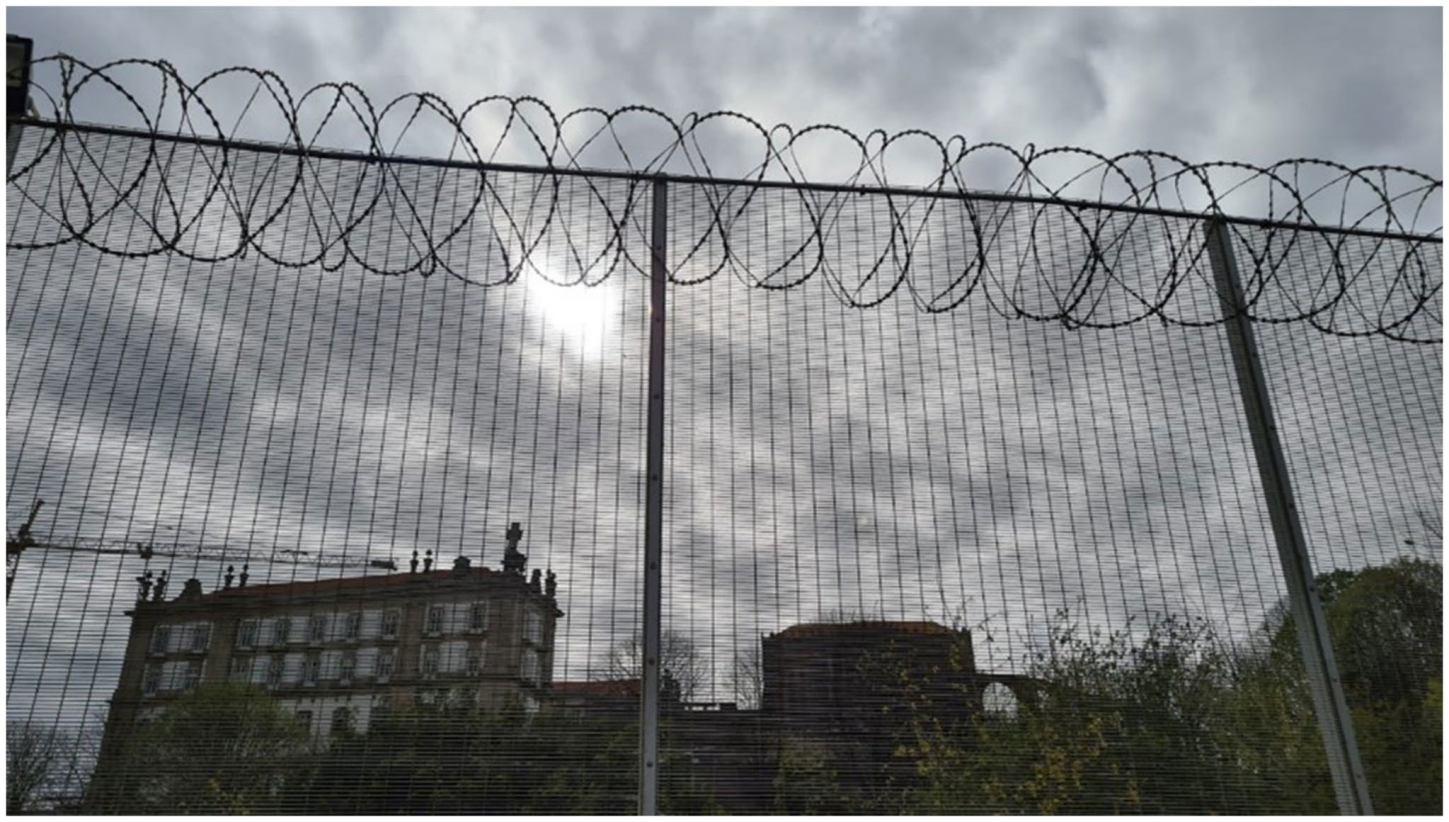
Santa Clara Juvenile Detention Center—Vila do Conde/Portugal (Caruso, 2022).

In 2020, Portugal registered at least 4,998 young people in “conflict with the law”, but only 90 were in juvenile detention centers (78 male and 12 female participants) (DGRSP, 2020). We found a similar reality in 2022 when we carried out field research, which, broadly speaking, sought to answer the following question: “What masculinities and femininities are young people from Educational Centers in Portugal constructing?” To this end, quantitative and qualitative approaches were combined to understand “Why some and not others?” (de Sousa e Silva, 2003) and their life trajectory marked by entry into a juvenile detention center. This question, which seems to be centered on the individual trajectory of the subjects investigated, instead leads us to think about the structural and institutional recurrences that affect the lives of these young people, thus considering key dimensions such as family ties, educational trajectories, ethnic–racial relations, relations with the police, justice, and, more precisely, the impacts that COVID-19 has had on their recent paths (Moura et al., 2023) (see Figure 1).

The research project entitled X-MEN—Masculinities, Empathy, and Non-violence^2^ aimed to promote non-violent masculinities^3^ to build dialogical strategies that seek to reduce gender-based violence in the context of the application of juvenile justice through the construction of tools and strategies that can be used in juvenile detention spaces by professionals who work daily with this public, as well as serving as training and improvement material for these professionals themselves on the subject. The acronym X-MEN—which gives the project its name—is inspired by the famous superhero comic books. In this case, it is worth remembering that in these books, X-MEN are a subspecies of humans who fight for peace and equality between “normal” humans and mutants (different from the norm). This conflict is often compared to real-world experiences. For this reason, it was appropriate to represent the age and identity of the young people (Abramo, 1994) who were the subjects of the research since these superheroes have always represented diversity and difference, with powers that are accentuated in their adolescence, making them an interesting “model” for the analogy with young people in the context investigated. This is how the proposed project used the figure of the mutants in 2023, after the diagnostic study had been completed, to promote a series of workshops aimed at building knowledge with and for young people, as well as making them reflect on individual attitudes and behaviors based on rigid gender norms (Moura et al., 2023).

According to data from the Portuguese government’s Directorate-General for Reintegration and Prison Services (DGRSP),^4^ in March 2022 (the month in which we began our fieldwork), there were 121 young people serving internment measures, which can be of at least 03 regimes: (a) open—in which the young people live in the detention center, but attend training and socio-educational activities, preferably outside; (b) semi-open—in which the young people live and attend training and socio-educational activities inside the center itself and can eventually return home and; (c) closed^5^—the young people live and attend training and socio-educational activities in the center, with exits, always under supervision, strictly limited to fulfilling judicial obligations, meeting health needs or other exceptional reasons.

During the fieldwork, questionnaires were administered to more than 80% of the universe of young people in compliance with the measures in the three regimes described above, as well as 11 individual interviews (nine men and two women) and six focus groups where the ethnofictional experiments that will be analyzed in this article were produced.

## 3 Who are the young people in the juvenile detention center?

By offering a brief photographic overview of what we were able to capture during the research project, it was possible to see that the participants who answered the questionnaire were mostly between 16 and 17 years old: 90% self-declared male, 9% female, and 1% self-declared another sexual identity. The majority of the young people were born in Portugal. Still, there were also those born in Brazil, Cape Verde, Mozambique, and Angola, as well as other nationalities, in smaller numbers. At the time of their internment in the Educational Centers, the young people had resumed their studies. They generally attended the 7th, 8th, and 9th years of basic education, which shows that the overwhelming majority were clearly out of step with the school year.

The data point to an erratic school career, accompanied by an early entry into the world of work, in precarious activities linked to temporary services in construction and commerce, which, for the vast majority—coincide with the work activities carried out by their parents. We were struck by the fact that, in some cases, they openly declared that they “worked in drug trafficking” or theft when we asked them what they had worked or done as a trainee before.

Of all the young people who took part in the questionnaire, two-thirds had already been institutionalized, which means that we are dealing with young people who, from a very early age, are under the protection of the State because of their dangerous condition, as provided for in Portuguese law.^6^ Measures such as support for parents and placement in foster homes were the most commonly reported in the survey.

It is worth noting that of all the people interviewed, 82% were serving a closed detention order, the most restrictive of all.

In the cases observed, the triangulation between an irregular school trajectory, the recurrence of promotion and protection measures since childhood, and entry into the Educational Centers for internment measures, based on an act analogous to crimes of a serious nature, is evident. The life stories of these young people exemplify what the field of studies on youth, masculinities, and violence has been pointing out for decades: longitudinal histories of marginalization and intergenerational transmissions of violence (Taylor et al., 2016; International Men and Gender Equality Survey (IMAGES), 2011; among others).

## 4 Literature review: ethnofiction as a window to multiple worlds and perspectives

In addition to mapping data on schooling or the nature of the criminal acts that young people may have committed, it was essential to understand how they fit into the social world. What ideas, values, and affections do they share? For this reason, ethnographic experimentation inspired by ethnofiction emerged as the ethical-methodological premise of the research.

I will now explain how and why. Inspired by the work of Jean Rouch (Ribeiro, 2007), a French anthropologist and filmmaker who, over more than 50 years, has produced important studies on West Africa, using cinematographic language to construct dense ethnographies in which the actors interpret stories they have constructed and which deal with their own reality; ethnofiction has been gaining new readings, adaptations, and multiple uses in different empirical contexts (Ferraz, 2010; Coelho, 2015; Cucinotta, 2017; Pessuto, 2017; Dantas, 2022), such as the one we intend to explore in the case analyzed here.

First, Coelho (2015) proposes that ethnofiction can be an ambiguous category and, therefore, difficult to grasp, either in Rouch’s own conception or from the reading of those interested in defining it. Sjöberg (2008) points out the term was probably coined not by Rouch himself but within film criticism since his critics interpreted his work as oscillating between “ethnographic film,” “documentary,” and “fictional film.” For his part, Rouch always argued that trying to theoretically “frame” his approach would be a waste of time.

It is not a documentary that attempts to capture an observed reality. By the same token, it is not a melodrama the filmmakers dreamed up to titillate our emotions – These films are stories based on laboriously researched and carefully analyzed ethnography. In this way, Rouch uses creative license to “capture” the texture of an event and the ethos of lived experience. (Stoller, 1992, p. 143)

For the purposes of this work, the idea of ambiguity that ethnofiction embodies could be useful insofar as the young people at the Center of the study lived this issue in their bodies: They were simultaneously very young and vulnerable while at the same time having very dense life stories marked by the crudeness of the violence they practiced or suffered.

On the other hand, ambiguity was a relevant analytical key as the use of fictional stories served as an escape valve to talk about an imagined other who simultaneously had much of himself. Therefore, the invitation to dive into the life of the character João was a window that opened up to talk openly about what cannot be told, at the risk of publicly assuming acts of infraction that have not been accounted for by the juvenile justice system, for example. This fundamental ethical issue permeates the work of researchers who dedicate themselves to producing studies on young people in conflict with the law and who must preserve the identity of their interlocutors.

Like Eaton (1979), Rouch also argued that “fiction is the only way to penetrate reality – the means of sociology remain external” (p. 8). In this way, the reality fictionalized by the young people in the research revealed more than individual creativity in narrating. However, it explored the regularities of situations that crossed their life stories, guaranteeing, in this case, high value for the socio-anthropological reflections that we wish to undertake.

Thus, ethnofiction as a methodological resource starts from an initial story outline in which the subjects begin to improvise characters for themselves in front of a camera. There is, therefore, no pre-established script because what is at stake is recognizing that improvisation can be capable of revealing aspects that were hitherto difficult to access through the predominantly described methods of ethnographic activity (Coelho 2015, p. 13).

Sjöberg (2008) argues that ethnofiction can be understood as a creative practice of ethnographic research as it articulates the improvisation of the narratives that the interlocutors construct with what they actually think about the world. Thus, ethnofiction proposes a way of expressing aspects of their own social reality that would be difficult to capture or demonstrate in any other way. The author also argues that it is not important for ethnofiction to make a rigid distinction between fiction and objective fact.

The central point is this collaborative methodology’s encounter between fiction and reality. From this perspective of analysis, this blurs the boundaries between what is imagined and what is lived, demanding equal hermeneutic creativity from the researcher to bring out analyses that go beyond the singular and/or particular story of the research subjects to propose an understanding of the regularities, the standard scripts in de Gaulejac’s (1996) terms, that these life stories reveal.

When discussing the use of fiction as a methodology in qualitative research, it is essential to consider the significant contributions of Patricia Leavy, who has devoted herself, in various books and articles, to exploring the notion of social fiction as a valuable tool for translating empirical data in an accessible, direct, and engaging manner. Creating characters, settings, and situations that illustrate social themes within research emerges as an innovative approach to addressing sociologically complex issues present in the author’s analytical repertoire, such as gender identity, violence, social inequalities, and sexuality. Furthermore, the point of convergence between her proposal and this article’s presentation lies in her advocacy for an intersection between art and science. Leavy (2020) suggests that social fiction occupies this intersection, demonstrating that combining analytical rigor with narrative creativity can generate a deeper understanding of the social reality being investigated. Thus, her approach, which integrates the ethnographic method with art, is an essential source of inspiration for this work, which argues that fiction can be a powerful resource for accessing the universe of emotions, subjectivities, and lived experiences, as in the case of young people in juvenile detention centers.

## 5 Methodological procedures

As highlighted in the introduction to this article, the young people taking part in the research in the Juvenile Detention Centers in Portugal were invited to collectively produce João’s story based on the initial elements they were given. The initial fictional piece had aspects that were very close to the reality they were experiencing in terms of age since the character João was 15 years old and was serving a sentence in a closed regime, just like the research subjects. From then on, the young people were given complete creative freedom to construct the story. They had approximately 30 min to talk to each other without any intervention from the researchers accompanying the focus group and without the researchers giving any direction to the proposed story.

They were only asked to write the story they had imagined collectively on a sheet of paper so that at the end of the activity, they could choose one of their classmates to read it aloud.

As if in a game of mirrors in which they narrate about João talking about themselves, the young people began to construct the story by negotiating among themselves the terms and expressions they would use and the characters from João’s life who would appear. Somehow, as we will see in the next section, in all of the six fictional pieces constructed throughout the study, the young people tried to cover aspects related to childhood, family life, school, friendships, entering the Juvenile Detention Center, and the future prospects that the character João might have. In this case, narrating involved the collective exercise of negotiating between the young participants what to tell and how to tell it, so they could choose how to write it down. Therefore, the writing process is understood as part of the collective reflexivity effort proposed in this methodology.

## 6 Results and discussion: what they told us about João

**Figure 2:**
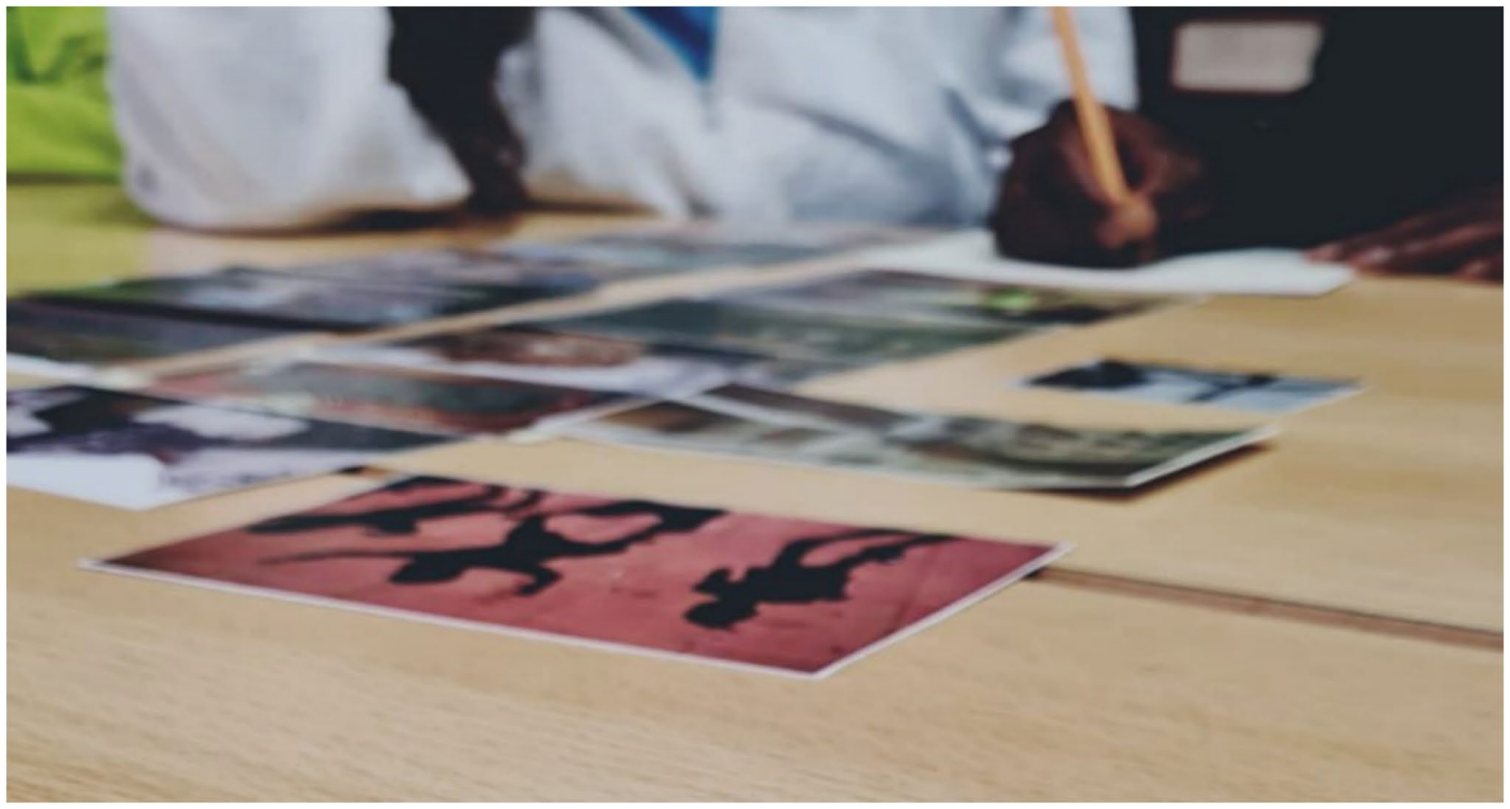
Building João’s story—image taken during the ethnofiction exercise in a youth focus group (Lisbon, March - Caruso, 2022).

In this section, we will return to the main themes explored by the young people in the study based on the ethnofiction exercise proposed. Stripping away their own life stories for a while to incorporate the stories of João, our fictional character, who was just as young as they were and was also imaginatively present in that Educational Center, was the strategy adopted so that they could talk together about this young man who was so similar to them, but who was none of them, giving them the creative freedom to put forward ideas and imagine the paths João would have traveled until he arrived at that Center (see Figure 2).

After sharing the stories, they could devote themselves to writing. The following lines present fragments of the stories they created, seeking to bring to the fore the way they write, the words they use, and how they express their ideas. They wanted to explore, first, which were the central points they chose to value in each story and, second, perceive recurrences and patterns in all the cases narrated.

### 6.1 Story 1—João, the caretaker of the brothers

“João had a good life before coming to the Educational Center. He liked being with his parents, but it wasn’t always easy, because his parents were in prison and he had to take care of his siblings (…) but to support them he had to rob some stores and restaurants, among other places.” (Center 03, March 2022)

The first story, constructed by young people from Center 3,^7^ explores the parental bonds interrupted by their parents’ imprisonment. It is one aspect several young people address in focus groups and interviews. One of the most frequently reported consequences, as in this version of the story starring João, is precisely the change in family roles as the older children take over from the parental figure of their younger siblings. Consequently, youngsters are already taking on caring responsibilities at a very early age. At the same time, the fact that João was looking for money to help raise his siblings was a motivating factor for him to take up robbery. What is striking is the theme of the precariousness of life. Several young people, both in the interviews and in the focus groups, told us about their desire to have things and about the family’s financial difficulties, especially as they were in families with more than three siblings where the mother and/or grandmother were often the sole breadwinners.

When João was sent to the Juvenile Detention Center for the theft he had committed, his brothers were left to go to a foster home. Therefore, the foster home-educational center continuum appears, once again, as a constant reality in the lives of young people in detention.

### 6.2 Story 2—João in his neighborhood

“He was disgusted with himself for the acts he had committed, but he did not feel sorry about it (…) He grew up in the 6 de Maio neighborhood, where everything happened, so he let himself be influenced by bad company.” (Center 5, March 2022)

The story told by young people from Center 5, one of Portugal’s mixed centers,^8^ brings to the fore life in so-called “clandestine” neighborhoods such as Bairro 6 de Maio,^9^ located in the Amadora area of Greater Lisbon.

The young people recount João’s anger at having been caught. Clearly, the discussion focuses not on whether João regrets what he did. Rather, they are concerned with highlighting the “neighborhood effect,” which translates into the influences of supposedly bad company that João has had along the way, which they point to as a justification for his path until he ends up serving a closed prison sentence for committing robberies. Once again, theft appears as a central and decisive issue for entry into Juvenile Detention Centers and the influence of neighborhood friends who encourage certain criminal practices. Another essential issue portrayed refers to the loneliness they feel when their emotional ties with friends and family are abruptly interrupted as a result of entering the Juvenile Detention Center.

### 6.3 Story 3—João who had a bad life

“Once upon a time, there was a 15-year-old boy who was born in a social district in Lisbon where there were many problems. Throughout his life, he did not go to school and lived a bad life. He had a family, but he did not care about them (…) he used to steal from gangs until he was caught red-handed…” (Center 2, March 2022)

On the other hand, the young people from Center 2 constructed João’s story, focusing on two issues that cut across all the youth dialogues we had throughout the study: erratic school trajectory and early entry into the world of criminal practices. This particular story, which focuses on “going through life in a bad way,” allows us, in some way, to understand how the young people themselves explain trajectories similar to their own: Thus, “going through life” means, in terms of João’s story, spending a lot of time on the streets to the detriment of family life. The streets are not just any streets in the city, but specific streets, geographically located in the neighborhoods, characterized as “social neighborhoods.”

However, the young people brought up the subject of gangs, which appeared in the study as a space for identity construction and affirmation but also as the link that allowed them to join robbery groups.

In this story, in particular, it is absolutely clear how the exercise of ethnofiction allows us to access the harrowing personal experiences of young people without them feeling exposed in their sharing. Let us see how, in the middle of the description, the voice of this other João shifts to that of himself when we detect the use of the 1st person singular to narrate the moment when “he was caught in the act, and I went to Caxias to be taken into custody.” In other words, in what would be an exercise in imagination, one of the young people recounts his experience serving a detention order at Centro Educativo 3.

Here, we see that the slip in assuming the first person in the supposedly fictional story implies unveiling a reality they commonly experience, such as arrests in the act of robbery.

### 6.4 Story 4—João was caught up in a raid in his neighborhood

“There was ‘rusgas’ in the neighborhood where two young men were caught…” (Center 4, April 2022)

This story tells of an important aspect in the lives of young people who have been to the Juvenile Detention Center: the relationship with the Police. In this case, the young people emphasize the native category “rusgas” which, in the conversations we had with them, translates the systematic police approaches in social neighborhoods, punctuated, in their accounts, as a crucial moment of crisis.

From a police perspective, these approaches aim to look for people involved in theft and trafficking. The neighborhoods considered in the Portuguese public debate to be “problematic” thus appear as a self-fulfilling prophecy, i.e., they are considered dangerous and therefore only “produce young delinquents.” The issue of social exclusion territories (Seixas and Guterres, 2018), which has an impact on the sociability dynamics of these young people, is something that deserves consideration since they are always more vulnerable to the networks of apprehension of the juvenile justice system, giving the false perception dominant in Portuguese society that only young people from these territories and with very defined ethnic–racial social markers “commit crimes.”

### 6.5 Story 5—I lost my dear grandfather

“João had been at the Educational Center for a few months. He grew up with his grandfather because his parents were in prison and his grandmother was ill, and João had to go and find work (…) One day, he went to rob the biggest hotel in his area and had to go and buy a gun. The contract he made was that he had to sell 15 kilos of powder, in other words, 15 kilos of cocaine. He accepted and started experimenting. By the time he managed to sell it, he was hooked and would not even stop at home until one day, and he received a message saying that his grandmother had died and he had to go and buy the powder to calm down. As he had no money, he went to rob the hotel. When he robbed it, he had to use his gun. He went to fire the gun and killed someone (…) And it’s over.” (Center 6, April 2022)

This story, constructed at Center 6, describes the character João, whose father figure is his grandfather, who raised him from a young age due to his parents’ imprisonment. What is striking about the story is the dynamics of arms and drug trafficking. Although João had already committed robberies, he decided to choose more ambitious targets, which meant he needed to buy a gun. As the gun was expensive and he did not have the means to buy one, he had to traffic drugs, which means that his entry into drug trafficking is a means to an end.

However, at a later point, the story also explores the dimension of drug addiction, which was mentioned so often in our conversations with the young people throughout the fieldwork.

So, João becomes addicted to cocaine. In one of the armed robberies, he commits murder. The spiral of violence recounted here in so few lines is concluded with a sentence that deserves to be highlighted because, at many moments, when we talked about future plans, the young people chose to remain silent, spoke in a reticent or even discouraged manner, as if they were telling us that it was impossible to think about the future… as the final sentence of this story denounces: And it’s over.

### 6.6 Story 6—Life has not smiled on him since he was a child

“Since he was a child, life had not smiled on him; his father was away because he was in prison, and there was no money in the house. His mother looked after João and two of his brothers. João grew up and realized the bad things happening in his life (…).” (Educational Center 01, April 2022) (Center 01, April 2022)

This story clearly demonstrates the emotional, social, and economic deprivation processes the character experienced.

“Life has not smiled on him since he was a child”, as spoken and written by young individuals between 14 and 17, is a crucial element for our reflection. Various factors contributed to this reality: the absence of a father who had been incarcerated—an experience that, as observed, is recurrent in the lives of these youths—as well as accounts of hunger endured by the protagonist, their siblings, and their mother. In some cases, they describe the father’s return after serving his prison sentence, while also exploring the recurring instances of domestic violence experienced by both the mother and her children.

This story brings multiple layers of analysis insofar as it synthesizes issues that marked the other stories constructed. It demonstrates how, in different research contexts (six centers spread across the country), with varied youth profiles coming geographically from different parts of Portugal, they manage to construct narratives that start from a journey from childhood to young life with similar elements.

One could be forgiven for thinking we are talking about excessively repetitive stories. Instead of doing so, we propose thinking that these recurrences, or rather, these accumulated outbreaks of violence, speak more of how the State, society, and families themselves deal with young people considered to be deviant to think about what windows of opportunity can be effectively built that can break the cycle of this violence that decisively mark young people’s trajectories and reproduce hegemonic masculinities and identities that are violent and undoubtedly destructive of these young people’s dreams.

## 7 Final considerations

This article deals with the methodological use of ethnofiction as a strategy for conducting fieldwork with young people in juvenile detention centers. It aims to construct a symbolic plot in which fiction and reality meet and thus give shape to the life stories that young people feel comfortable telling. Ethnofiction, therefore, emerges as a powerful reflexive resource for sharing what is not always possible to narrate in the first person.

From the stories constructed by the young people explored here, it was possible to get to know the character João in his multiple dimensions such as childhood, adolescence, school life, interrupted and precarious family ties, community and friendship ties, the dynamics of his criminal practices, his relationship with the Police, his entry into the Centers, and, finally, the difficulty in thinking about a possible future.

Through the ethnofiction proposed, the experiences of our interlocutors emerged while respecting the silences, shame, fantasies, and desires of young people who are so young but who already bear the deep scars of a precarious and suspended life.

The theoretical-methodological proposal of shared anthropology that is ethically committed to the research interlocutors, as suggested by Jean Rouch, was the guiding thread for the field experience carried out in six juvenile detention centers in Portugal throughout 2022.

In this way, I argue that using fiction as a language to access the social worlds lived by the young people researched proved methodologically powerful. First, it was possible to escape the classic research scripts with this target audience, which generally values the deviant nature of their actions much more than the political, economic, socio-cultural, and affective context that their life experiences reveal.

In this shared practice of producing knowledge about themselves and the world around them, they could understand—in their own terms—the nuances of a young life that was, at the time, characterized by freedom restriction.

## Data Availability

The datasets presented in this study can be found in online repositories. The names of the repository/repositories and accession number(s) can be found at: https://xmen.ces.uc.pt/.

## Footnotes

^1^Tatiana Moura (CES-UC) coordinated the research, which also included researchers Marta Mascarenhas and Tiago Rolino (CES-UC) and the author of this article.

^2^The X-MEN Project—Masculinities, Empathy, and Non-Violence began in 2022 and will end in June 2024. It was funded by the European Commission‘s Citizens, Equality, Rights and Values Program (CERV) (Grant Agreement No. 101049457). In addition to Portugal, the project also involved the CEPAIM Foundation from Spain and the Status M non-governmental organization from Croatia. The results of the project can be accessed at: https://xmen.ces.uc.pt/.

^3^In Portugal, the promotion of non-violent masculinity was included as an indicator in the National Action Plan for Equality between Women and Men (PAIMH) of the National Strategy for Equality and Non-Discrimination [ENIND] 2018–2030 (approved by Resolution of the Council of Ministers No. 61/2018, of May 21).

^4^Monthly statistics for juvenile detention centers (March 2022). Available at: https://dgrsp.justica.gov.pt/Estat%C3%ADsticas-e-indicadores/Centros-Educativos, accessed December 20, 2022.

^5^The application of the closed regime of execution of the MTE takes place when the criminal offense committed corresponds to a maximum prison sentence of more than 5 years or when the minor is more than 14  years old at the time of the application of the measure and, cumulatively, the commission of two or more facts qualified as crimes against persons with maximum penalties of more than 3 years is involved.

^6^Law no. 147/1999, of September 1, republished by Law no. 23/2017, of May 23 (most recent version)—Law for the Protection of Children and Young People in Danger (LPCJP).

^7^To guarantee the anonymity of the research subjects and the places where they were serving a detention order, all the juvenile detention centers mentioned will only be numbered, thus preserving their identities.

^8^Mixed center serves young men and women.

^9^The so-called Bairro 6 de Maio is an emblematic case in the Portuguese context. From a neighborhood that was self-built by many Cape Verdeans, stimulated by the possibility of working in construction during the post-revolution period, to its near extinction 30 years later through government rehousing programs, many stories of pain and struggle mark those who were (and still are) in this territory that mixes belonging, survival strategies, resistance, and exclusion. For more information, see https://sinalaberto.pt/bairro-6-de-maio-a-luta-ininterrupta-de-uma-comunidade/, accessed March 26, 2023.
